# Ecological Momentary Assessment versus Weekly Questionnaire Assessment of Change in Depression

**DOI:** 10.1155/2024/9191823

**Published:** 2024-07-11

**Authors:** Jeanette Tamm, Keisuke Takano, Leah Just, Thomas Ehring, Tabea Rosenkranz, Johannes Kopf-Beck

**Affiliations:** ^1^Max-Planck-Institute of Psychiatry, Munich, Germany; ^2^Department of Psychology, LMU Munich, Munich, Germany; ^3^Human Informatics and Interaction Research Institute (HIIRI), National Institute of Advanced Industrial Science and Technology (AIST), Tsukuba, Japan; ^4^DZPG (German Center for Mental Health), Munich, Germany

## Abstract

**Objective:**

Ecological momentary assessment (EMA) is increasingly used to monitor depressive symptoms in clinical trials, but little is known about the comparability of its outcomes to those of clinical interviews and questionnaires. In our study, we administered EMA and questionnaires to measure change in depressive symptoms and repetitive negative thinking (RNT) in a clinical trial and investigated (a) the size of intervention effects associated with both techniques and (b) their validity in predicting clinical interview outcomes (i.e., global functioning).

**Materials and Methods:**

Seventy-one depressed patients were randomly assigned to one of three psychological interventions. The EMA comprised a concise item set (four items per scale) and was administered three times per day during a 7-week intervention period. Conversely, questionnaires were assessed weekly (WQA), encompassing their full sets of items of depressive symptoms and RNT.

**Results:**

While EMA excelled in detecting significant intervention effects, WQA demonstrated greater strength in predicting clinician ratings of global functioning. Additionally, we observed significant differences in time effects (slopes) between the two techniques. WQA scores decreased steeper over time and were more extreme, e.g., higher at baseline and lower postintervention, than EMA scores.

**Conclusions:**

Although clinical interviews, questionnaires, and EMA outcomes are related, they assess changes in depression differently. EMA may be more sensitive to intervention effects, but all three methods harbor potential bias, raising validity and reliability questions. Therefore, to enhance the validity and reliability of clinical trial assessments, we emphasize the importance of EMA approaches that combine subjective self-reports with objectively measured behavioral markers. This trial is registered with osf.io/9fuhn.

## 1. Introduction

Accurate reporting of changes in depression is essential for symptom monitoring and research on the effectiveness of interventions. Retrospective questionnaires, commonly used for this purpose, are usually administered pre- and post-intervention or on a weekly basis. Consequently, they average symptom severity based on patients' recall over the past week or weeks [1, 2]. Despite their common use, the validity of retrospective questionnaires is questionable. Depression is a dynamic disorder with large symptom fluctuations over time [3]. Particularly, depressed mood and processes of depression such as repetitive negative thinking (RNT) are known to be highly variable within days and across multiple days [4, 5, 6]. RNT describes the cognitive process of recurrent dwelling on negative content, often experienced as intrusive and challenging to control. It includes worry and rumination and is regarded as a transdiagnostic process that plays a central role in the development and maintenance of emotional disorders [7]. Given the pronounced fluctuations in symptoms and associated processes like mood and RNT, retrospective measurement is challenging. The reason lies in the human memory of emotional experiences, which is distorted, especially in depression [8]. Numerous studies have investigated that people tend to overestimate their experiences of positive and negative affect when asked to recall them retrospectively [9, 10, 11, 12], and in depression this recall bias is further amplified, particularly concerning negative affect [13, 14].

An alternative assessment technique that prevents recall bias is ecological momentary assessment (EMA) [15, 16, 17]. Conducted on smartphones, EMA takes place in patients' daily lives and allows repeated sampling of psychological states such as feelings, thoughts, or behavior in the moment [18]. Although EMA and other time-series-based procedures are increasingly used in clinical research, the practice of implementing them into clinical trials is not widespread [19]. Moore et al. [20] and Targum et al. [21] were the first clinical trials tracking depressive symptoms with EMA. Moore et al. [20] examined the intervention effects of mindfulness therapy on depression, mindfulness, and anxiety using EMA administered 10 days before and after the intervention compared to point-assessments with questionnaires. They investigated that EMA was associated with more pronounced intervention effects for mindfulness and depression. Moreover, the number needed to treat (NNT) was 45%–74% lower for the EMA compared to the questionnaire assessments. The authors conclude that EMA may be more sensitive in detecting and quantifying intervention effects due to its avoidance of recall bias inherent in retrospective questionnaires. Additionally, Targum et al. [21] discovered that change in depression assessed with EMA continuously over a 6-week antidepressants trial predicted change in depression rated by clinicians pre- and post-intervention.

Previous studies showed that EMA is an efficient and valid assessment technique to assess change in depression and they support the hypothesis that EMA is more sensitive to this change than point assessments with questionnaires. However, to establish EMA as an alternative assessment technique to questionnaires in clinical trials, further investigations are needed. Questions that need more research are: How comparable are EMA and questionnaires assessments (a) when questionnaires are administered weekly instead of just before and after the intervention, (b) when the EMA is more brief than the questionnaire, which is typically a need in order to reduce participants' burden when responding to several occasions per day, and (c) when comparing their validity in predicting global intervention outcomes rated by clinicians, such as global functioning?

In this study, we measured change in depressive symptoms and RNT with EMA continuously over the course of a 7-week clinical trial in comparison to weekly questionnaire assessments (WQA). The aim was to investigate two different aspects. First, our aim was to replicate the findings of Moore et al. [20] by testing whether EMA demonstrates larger intervention effects in the comparison of two different intervention conditions than questionnaires. In line with Moore et al. [20], we hypothesized that change in depressive symptoms and RNT assessed with EMA would be associated with larger intervention effects. Second, we investigated differences between the two assessment techniques in predicting change in global functioning rated by clinicians. We hypothesized that, after controlling for baseline global functioning, changes in depressive symptoms and RNT assessed with EMA would predict post-intervention global functioning more strongly than the same predictors assessed with WQA. For our analysis, we used data from the OPTIMA study [22], a clinical trial investigating the effectiveness of three different psychological interventions for depression: schema therapy (ST) versus individual supportive therapy (IST) and cognitive behavioral therapy (CBT) administered to moderately to severely depressed patients in an inpatient and day clinic setting.

## 2. Materials and Methods

### 2.1. Participants

To design our sample size, we performed computer simulations to detect a significant interaction between time and the intervention conditions with multilevel models (MLM). We based these simulations on the findings of Moore et al. [20], where the mindfulness-based stress reduction intervention had an effect of Cohen's *d* = 0.4 on the mindfulness outcome at the postintervention assessment. The results of our simulations indicated that the required sample size is around 20 per condition (resulting in 60 patients in total) to achieve a power of 0.80 under alpha = 0.05. We calculated a mean dropout rate of 20% (experiences of the OPTIMA study) and additional 20% due to insufficient EMA data, so we aimed a sample size of *n* = 33 patients per intervention condition.

Dropouts during the conduct of the study were defined as enrolled patients who were found to have incorrect in- or exclusion criteria during the conduct of the study, who left the clinic before end of intervention, or who missed more than six sessions (22%) of their intervention. Survival analyses were conducted to test for differences in dropout risk between our intervention conditions.

### 2.2. Design and Procedures

We analyzed data collected as part of the OPTIMA study [22] (identifier on clinicaltrials.gov: NCT03287362), a monocentric, rater-blinded, prospective, parallel-group, block-randomized clinical trial with repeated measures, and three intervention conditions (CBT, ST, and IST). The OPTIMA study was conducted at the Max Planck Institute of Psychiatry in Munich, Germany.

Inclusion criteria were age 18–65 years, having a compatible smartphone and a diagnosis of a major depressive disorder, single episode or recurrent, moderate or severe without psychotic symptoms diagnosed by clinical assessment. Patients received an expense allowance based on the response rate they achieved in the EMA. The study protocol was approved by the Institutional Ethics Committee of the Faculty of Medicine at LMU Munich (Project number 17–395). All participants provided written informed consent prior to clinical interviews, further measures, and randomization. More detailed information about exclusion criteria and further procedures of the OPTIMA trial are given elsewhere [22].

### 2.3. Interventions

Patients enrolled in the OPTIMA study were randomly allocated to one of three intervention conditions (schema therapy (ST), individual supportive therapy (IST), or cognitive behavioral therapy (CBT)), each one lasting 7 weeks and consisting of two individuals (50 min each) and two group (100 min each) sessions per week. Details about the different interventions are described in the OPTIMA study [22]. CBT, which is based on Beck's theory of depression [23, 24], is recommended as first-line psychological intervention for depression [25]. In contrast, ST is a transdiagnostic psychological intervention that is mainly rooted in cognitive therapy but integrates techniques of different therapeutic approaches such as psychodynamic therapy, gestalt therapy, and ergotherapy [26]. IST was used as an active and nonspecific approach that follows the concept of a bio-psycho-social disease model of depression and is based on the common factors of psychotherapy [27, 28, 29].

### 2.4. Concomitant Care

The OPTIMA study design did not regulate parallel psychopharmacotherapy or potential influencing factors inherent to an inpatient or day clinic intervention program, such as ergotherapy or case management. Decisions hereon were left to the psychiatrist in charge. To mitigate biases, all concomitant care was documented for subsequent use as potential confounders in the statistical analysis.

### 2.5. Measures

Comprehensive information regarding all measures conducted in the OPTIMA trial, encompassing various questionnaires, clinical interviews, imaging, and tests, is available in the OPTIMA study [22]. Only the measures of the EMA substudy relevant to our analysis are described here. These include five primary outcome variables: questionnaire and EMA scores of depressive symptoms and RNT and global functioning measured with a clinical interview pre- and post-intervention. Additionally, a short feasibility questionnaire of the EMA was assessed at the end of the intervention. Table S1 provides an overview of the assessment plan of the study.

#### 2.5.1. Ecological Momentary Assessment

EMA was conducted continuously throughout the entire intervention period, starting directly after patients' enrollment in the study. It comprised three prompts per day and was signal contingent, i.e., patients were automatically prompted by their device. Each day was divided into three phases (morning, noon, and evening). When installing the EMA app, participants reported their approximate wake-up time. The times of the three phases were based on this approximate wake-up time (morning: 2 hr before–5 hr after approximate wake-up time; noon: 5–10 hr after the approximate wake-up time; evening: 10 hr after–2 hr before the approximate wake-up time). During each phase, patients received one EMA prompt, which could only be completed within its assigned phase. Once a phase was over, the prompt could no longer be completed, but the subsequent phase's prompt was provided for participants to respond. Additionally, patients received semirandomized reminder to complete the EMA prompts. The mean time between patient responses was *M* = 303.8 min (SD = 94.06 min) (morning to noon), *M* = 337.8 min (SD = 106.66 min) (noon to evening) and *M* = 810.6 min (SD = 110.03 min) (evening to morning). This suggests that the randomization procedure successfully prevented temporal clustering of responses. To control for sequence effects, the EMA item order was randomized across prompts. Including baseline, this resulted in a total of 168 (56 days × 3 prompt per day) EMA prompts.

The EMA score of depressive symptoms was calculated by summing four EMA items, which were developed by the first and the last author in consultation with clinicians and based on diagnostic criteria for major depression (ICD-10). The four items represent three core symptoms of depression (loss of interest, withdrawal, and psychomotor agitation/inhibition) and current mood.

The EMA score of RNT was recently developed by another study [30] and has shown excellent model fit, high reliability, and good validity with depression outcomes. The paradigm comprises four EMA items. Three of the items are from the Perseverative Thinking Questionnaire (PTQ) representing the core characteristics of RNT (repetitiveness, intrusiveness, and uncontrollability). The fourth item measures subjective burden through RNT. Detailed information on the specific wordings of all EMA items used in this study can be found in Table S2.

We computed internal reliabilities for both EMA total scores (depression and RNT) using the “multilevel.reliability()” function in the “psych” package of R [31, 32, 33]. This function calculates both within-participant reliability of change over time points (i.e., Rc) and between-participant reliability of the averaged scores over *k* number of time points (i.e., RkF). For both, depressive symptoms and RNT, we observed good within participant reliability (depressive symptoms: Rc = 0.79, RNT: Rc = 0.86) and excellent between participant reliability (depressive symptoms: RkF = 0.1, RNT: RkF = 0.1).

The response scale of all EMA items, except for the mood item, was two-stepped: participants responded to a binary Yes–No scale (i.e., the same negative thoughts keep going through my mind again and again, Yes or No). If Yes was selected, a five-point Likert scale followed, which assessed the extent of agreement (labeling: not at all, a bit, moderately, considerably, very much). In contrast, if No was selected, the Likert scale did not appear, and the next item followed. Participants rated their mood by selecting one of five emojis (labeling: very good, good, moderate, bad, and very bad) that best described their current mood.

#### 2.5.2. Weekly Questionnaire Assessments

The corresponding questionnaire scores of depressive symptoms and RNT were assessed weekly, resulting in a total of eight assessment points, including baseline. Depressive symptoms were assessed with the Beck Depression Inventory-II (BDI-II) [1], a widely used self-rating instrument that accounts for different depressive symptoms. RNT was assessed with the Perseverative Thinking Questionnaire (PTQ) [34], which evaluated three core characteristics of RNT: repetitiveness, intrusiveness, and the difficulty in disengaging from negative thoughts.

#### 2.5.3. Clinical Interviews

Clinical interviews were conducted to assess patients' global functioning. Trained and blinded raters employed the World Health Organization Disability Assessment Schedule (WHO-DAS) [35]. In the OPTIMA study, interrater reliability (intraclass coefficient) was routinely assessed, showing excellent agreement (*M* = 0.998, SD = 0.004) [22].

### 2.6. Statistical Analyses

As outlined in our preregistered analysis, we planned to filter patients with an EMA response rate below 33%. Our intention was to align with similar approaches found in the literature [20]. However, new methodological recommendations [36] suggest setting such arbitrary cutoffs lowers statistical power. Therefore, we decided to deviate from the preregistered protocol, i.e., to include all patients in the analyses regardless of their response rate. As a sensitivity analysis, we repeated the same analyses with the planned cutoff (i.e., to omit data from patients with a response rate <33%), and we found that the results were unchanged. As planned, however, we checked person-level standard deviations for each EMA item across the trial period. For plausibility, patients with a standard deviation of zero in at least one EMA item were excluded. Additionally, we recognized some prompts with missing items, which we filtered before running the formal analyses.

Differences between intervention conditions in demographics, baseline variables, and response rates were examined using *χ*^2^ tests for nonparametric variables and ANOVA tests for parametric variables. Baseline depression, gender, response rates in EMA and WQA, intervention condition, and concomitant care were included as covariates in our statistical models. Our primary variables were standardized using individual person's means and standard deviations, allowing for a conversion of EMA and questionnaire values into a common unit. In all our statistical analyses, a *p*-value less than 0.05 was considered significant.

#### 2.6.1. Hypothesis 1

To test our first hypothesis, we estimated four parallel MLMs, separately for depressive symptoms or RNT assessed with EMA or WQA. The MLMs were implemented using the R package “nlme” [37]. MLMs accommodate the nested structure of the data, with occasions (level 1) nested within individuals (level 2). An alternative three-level data structure with occasions nested within days nested within individuals was considered but rejected to keep model complexity low. Adding the day level into a three-level model explained less than 2% of additional variance (intraclass correlation (ICC) of day in depressive symptoms: 1.6%, ICC of day in RNT: 1.97%). The two-level MLMs were specified as follows:

(1) $$\begin{array}{c} Y_{ij}={\text{int}}_{j}+{\text{slp}}_{j}\times {\text{time}}_{ij}+r_{ij}, \end{array}$$

(2) $$\begin{array}{c} {\text{int}}_{j}=\gamma_{00}+\gamma_{01}\left(\text{intervention }{\text{condition}}_{j}\right)+u_{0j}, \end{array}$$

(3) $$\begin{array}{c} {\text{slp}}_{j}=\gamma_{10}+\gamma_{11}\left({\text{intervention condition}}_{j}\right)+u_{1j}, \end{array}$$

where *Y*_*ij*_ is the EMA or WQA-assessed levels of depressive symptoms or RNT of the *j*th participant at time *i*. The residual is denoted by *r*_*ij*_. Note that the unit of time was different between the EMA and WQA models (moment vs. week). Both intercepts (int_*j*_) and slopes (slp_*j*_) were allowed to vary across individuals (random effects). Individual differences in the intercepts and slopes were explained by the intervention conditions, which were explicitly assumed as fixed effects (*γ*_01_ and *γ*_11_). The effect of the intervention conditions on the slope (i.e., *γ*_11_, time–condition interaction) was of our particular interest, which represents how the change rate in an outcome differed across the three intervention conditions.

To obtain standardized intervention effects, we calculated Cohen's *d* [38] and the NNT [39]. Cohen's *d* effect sizes were calculated for individual slopes (slp_*j*_) using the “cohensD()” function from the R package “lsr” [40]. The NNT index represents the number of individuals who need to undergo treatment for one person to benefit from the intervention compared to an alternative intervention or a control condition. A lower NNT is considered favorable as it implies a higher likelihood of benefitting from the intervention. We calculated the NNT using the function “NNT()” from the R package “dmetar” [41], assuming that 44% (reference group: IST) or 46% (reference group: CBT) of the reference condition would respond to the intervention. We defined intervention response as a 50% decrease in BDI-II score from pre- to post-intervention, which is a common definition used in depression literature [42].

#### 2.6.2. Hypothesis 2

The second hypothesis was tested by submitting the individual change rates (i.e., slopes of time) from the MLMs of Hypothesis 1 into multiple regression models that predicted patients' global functioning postintervention. Again, we ran four parallel models, one for each slope derived from the MLMs of EMA- or WQA-assessed depressive symptoms or RNT, serving as the focal predictors of the models.

Additionally, we estimated two combined models including both assessment techniques simultaneously. In all models, we controlled for the baseline levels of global functioning. The separate models were conducted to ascertain if both EMA and WQA slopes significantly predicted postintervention global functioning independently, and the combined models determined the relative strength of both predictors.

In the last step, we conducted model comparisons to assess variations in the explained variance between the separate and combined prediction models. Specifically, we employed two widely recognized information criteria, namely the Akaike information criterion (AIC) and the Bayesian information criterion (BIC) [43].

#### 2.6.3. Additional Analysis

To explore the distinctions between EMA and WQA more deeply, we performed an additional analysis to our preregistered analysis plan, investigating whether time effects (slopes) significantly differed between the two assessment techniques. To achieve this, we aggregated the EMA scores of depressive symptoms and RNT to weekly means and conducted MLMs, including “assessment technique” (EMA vs. WQA) as a predictor. We conducted two separate models, one with depressive symptoms and one with RNT as the dependent variable. The models included occasions and assessment techniques as fixed effects, while considering patients as random effects to account for individual variability. Our primary focus was the assessment technique-by-time interaction as the fixed effect of interest. Given that our EMA and WQA scores predominantly comprised different items, we also conducted corresponding MLMs at the level of three individual RNT items that were identical between the EMA and WQA scores of RNT.

### 2.7. Transparency and Oppenness

We report how we determined our sample size and all manipulations in the study, and we follow JARS [44]. Here we reported only the data exclusions and measures relevant to the conducted analyses. All data exclusions and measures are described in the study protocol of the OPTIMA trial [22], of which this study is a substudy. The dataset generated and analyzed for this study contains clinical data and is not publicly available due to the protection of participants' rights to privacy and data protection, but is available from the corresponding author upon reasonable request. Materials and analysis code for this study are available by emailing the corresponding author. This study's design, hypotheses, and analysis plan was preregistered before the end of the data collection and before analyses were undertaken; see osf.io/9fuhn. Data were analyzed using R, version 4.2.2 [45].

## 3. Results

### 3.1. Sample Description

**Figure 1:**
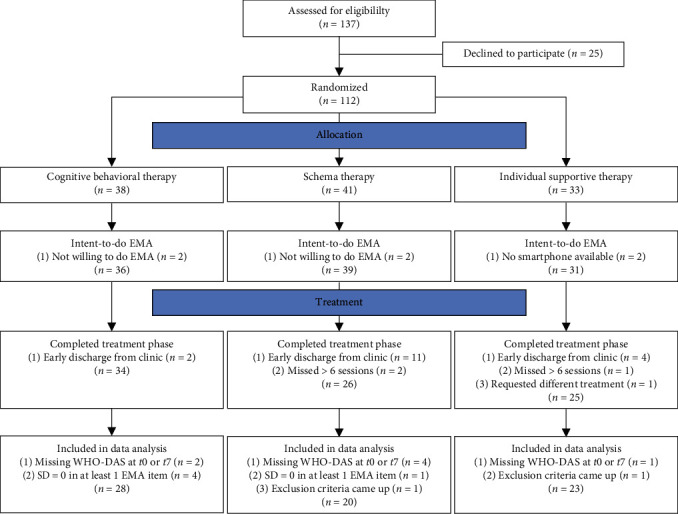
Data exclusion flow diagram. *Note*. EMA, ecological momentary assessment; WHO-DAS, World Health Organization-Disability Assessment Schedule.

The study was conducted from August 2019 to December 2020. Initially, 137 patients were recruited and assessed for eligibility, of which 106 met inclusion criteria, expressed willingness to participate in the EMA, and were randomly assigned to the intervention trial (ST, 39, CBT, 36, IST, 31). Detailed information is presented in the CONSORT flowchart in Figure 1. Survival analyses (Figure S1) revealed that patients in CBT exhibited a significantly lower risk of dropping out during the intervention phase compared to those in ST (Cox regression: *ß* = −1.70, *p* = 0.02; relative risk in CBT = 0.17, 95% confidence interval (CI) = 0.04–0.75). IST did not significantly differ from ST (*p* = 0.2) or CBT (*p* = 0.66) regarding dropout risk. Reasons for dropout included early discharge from the clinic before completion of the intervention trial (17), missing more than six intervention sessions (3), and request for a different intervention (1). Before analyses, we further excluded the data of 14 patients due to ineligible diagnoses that surfaced during the study (2), patients that missed one of the clinical interviews (7) or lack of conscientious completion of the EMA (5), which was defined as a standard deviation of zero in at least one EMA item during the intervention trial. Ultimately, data of 71 patients (CBT, 28, ST, 20, IST, 23) were included in the statistical analyses.

Descriptives of the study sample are presented in Table 1 and Table S3. At baseline, patients exhibited severe levels of depression on average (BDI-II: *M* = 32.57, SD = 8.4). As shown in Table 1, patients in the intervention conditions exhibited significant differences in baseline depression and gender. Specifically, holm-adjusted post-hoc *t*-tests between conditions revealed a significant difference between CBT and the two comparing intervention conditions IST and ST in baseline BDI-II (CBT: *M* = 29, SD = 7.35; IST: *M* = 35.83, SD = 8.62; ST: *M* = 33.8, SD = 7.89; CBT–IST: *p* = 0.004; CBT–ST: *p* = 0.037). Consequently, depression severity at baseline and gender were included as additional covariates in the analyses.

In the analysis sample, patients responded to a mean of 55.77% (SD = 25.31%) of the EMA prompts (Table 1). We found significant differences in the distribution of patients response rates over the intervention weeks (*F* (7, 560) = 4.72, *p* < 0.001), displayed in Figure S2. Note that patients had in mean 0.69 (min = 0, max = 11) prompts with missing items. EMA prompts with missing items (*N* = 49) were filtered out of the analysis dataset. The EMA feasibility questionnaire underscored the good acceptance of the EMA among patients, with 92.73% expressing a liking for our app—rating it as very good, good, or reasonably good.

### 3.2. Hypothesis 1

To test whether change in depressive symptoms or RNT assessed with EMA is associated with larger intervention effects than when assessed with WQA, we ran MLMs separately for depressive symptoms or RNT measured with EMA or WQA and calculated standardized effect sizes. For each MLM, we compared a simple model including only time and intervention condition as predictors with a complex model including gender, baseline depression, response rate, and concomitant care as covariates. We compared the simple and the complex models with model comparison analyses (Table S4). As the complex models explained our data not significantly better than the simple models and no covariates were significant predictors, we reported the results of the simple models and also based further analyses on them (Table 2). In the MLM analyzing EMA-assessed depressive symptoms, and the MLMs for the two WQA scores (RNT and depressive symptoms), we observed significant time predictors, indicating substantial score changes from baseline to the end of the intervention. Moreover, we identified a significant intervention effect, denoted by a significant time × condition interaction, for the EMA-assessed RNT when comparing ST with both reference intervention conditions, IST and CBT. Furthermore, when looking at the standardized effect sizes between ST and the reference interventions, Cohen's *d* effect sizes were higher, and the NNTs (to detect an additional responder in ST versus IST and CBT) were lower for EMA-assessed than WQA-assessed RNT (Table 3).

### 3.3. Hypothesis 2

Multiple regression analyses were conducted to test whether change in depressive symptoms or RNT assessed with EMA predicts change in global functioning measured by clinician ratings more strongly than the same predictors assessed with WQA (Table 4). We ran four separate models, one for each slope of EMA- or WQA-assessed depressive symptoms or RNT as the focal predictor. Additionally, we performed two combined models (one for depressive symptoms and one for RNT) including both assessment techniques as predictors (EMA and WQA). The separate models revealed that both EMA-assessed and WQA-assessed slopes of depressive symptoms, along with the WQA-assessed slope of RNT, significantly predicted post-intervention global functioning, while the EMA-assessed slope of RNT stayed hovered just below the significance threshold (*p* = 0.051). In the combined models, none of the EMA slopes significantly predicted our dependent variable, whereas both WQA slopes did. We conducted model comparison analyses to test the combined models including both predictors (EMA and WQA) against the separate WQA models. The AIC and BIC values of the simple models were slightly lower than those of the complex models (AIC/BIC for simple models: depressive Symptoms—83.29/92.35, RNT—85.45/94.5; AIC/BIC for complex models: depressive Symptoms—83.1/94.41, RNT—87.43/98.74), indicating no significant improvement in model fit with EMA slopes as additional predictors.

### 3.4. Additional Analysis

**Figure 2:**
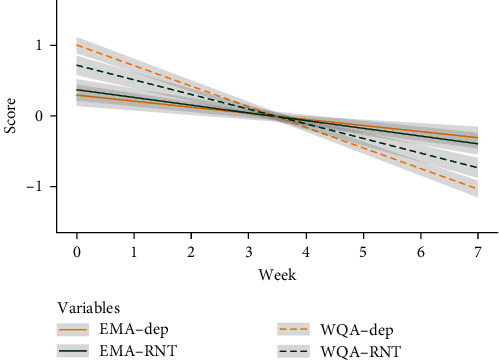
Comparison of EMA versus WQA data in the time series. *Note*. The figure illustrates the linear modeling of the reduction in patients' depressive symptoms (orange) and RNT (green) measured with EMA (solid lines) versus WQA (dashed lines) during the 7-week intervention period. All scores are *z*-standardized and person mean centered. EMA scores are aggregated to weekly means and 95% confidence intervals are shown (light gray). EMA, ecological momentary assessment; WQA, weekly questionnaire assessment; and RNT, repetitive negative thinking.

Figure 2 illustrates the linear modeling of the reduction in patients' depressive symptoms and RNT, as assessed by EMA and WQA, throughout the 7-week intervention period (please note that the scores are person-mean centered and *z*-standardized). To dive deeper into the distinctions between EMA and WQA, we conducted two further MLMs (one for depressive symptoms and one for RNT), including “assessment technique” (EMA vs. WQA) as a predictor (Table 5). In both models, we found significant time × assessment technique interactions, revealing significant differences in time effects (slopes) between EMA and WQA. Given that our EMA and WQA scores predominantly comprised different items, we also ran corresponding MLMs at the level of three individual RNT items that were identical between the EMA and WQA scores of RNT. At this item level, the results were similar to those obtained with the full-item score of RNT.

## 4. Discussion

This study compared two different techniques, EMA and WQA, to measure change in depressive symptoms and RNT within a clinical trial. The primary objectives were twofold: (a) to determine their sensitivity in detecting intervention effects between conditions and (b) to assess their validity regarding the prediction of clinician-rated global functioning. The study underscores the feasibility of continuous EMA throughout the whole course of a clinical trial in moderately to severely depressed patients. To the best of our knowledge, it is the first clinical trial studying this technique in comparison to a questionnaire setup that is usual in clinical trials: weekly assessment of full questionnaires.

### 4.1. Intervention Effects

In a study investigating the effects of mindfulness therapy on mindfulness, depression, and anxiety, Moore et al. [20] identified higher intervention effects for EMA-assessed mindfulness and depression compared to questionnaire-assessed measures. This indicates a heightened sensitivity of EMA in detecting significant intervention effects. Our results support this assumption. Based on these findings by Moore et al. [20], we aimed to examine whether changes in depressive symptoms and RNT assessed with EMA would result in larger intervention effects than those assessed with WQA. Indeed, our analysis revealed that EMA-assessed RNT is associated with significant intervention effects, accompanied by higher effect sizes and lower NNTs compared to the WQA variable. Notably, contrary to our initial hypothesis, we observed this effect only for RNT, while for change in depressive symptom we found no significant intervention effects, regardless of whether they were assessed with EMA or WQA. However, this does not undermine the underlying assumption of EMA's heightened sensitivity in detecting such effects. Interestingly, our findings for depressive symptoms align with the results of the OPTIMA study [22], of which our study is a substudy. Including a larger population of 292 patients, even the OPTIMA study [22] found no significant intervention effects on depressive symptoms between ST and the two comparing interventions, CBT and IST. This supports the notion that ST is indeed comparably effective in reducing depressive symptoms when compared to CBT and the active control condition IST and serves as a plausible explanation for why not even EMA detected intervention effects between these conditions for changes in depressive symptoms in our study.

### 4.2. Prediction of Change in Global Functioning

Studying the effects of a 6-week antidepresssant trial, Targum et al. [21] discovered that continuous EMA of changes in depression significantly predicted clinician-rated outcomes. To corroborate these findings, we analyzed the validity of EMA and WQA in predicting changes in global functioning. We examined them in separate models (EMA or WQA) and in combined models incorporating both predictors (EMA and WQA). Changes in depressive symptoms and RNT assessed with WQA significantly predicted changes in global functioning in both the separate and combined models. In contrast, the results for EMA were mixed. Specifically, changes in depressive symptoms assessed with EMA emerged as a significant predictor in the separate model, but not in the combined model, and EMA-assessed changes in RNT failed to significantly predict change in global functioning in either the separate or combined model. The results are consistent with those of Targum et al. [21], in that EMA-assessed changes in depression significantly predict clinician ratings. Nevertheless, our hypothesis, asserting that EMA-assessed changes in depressive symptoms and RNT could be stronger predictors of clinician-rated changes in global functioning, was not supported by the results. A plausible explanation for our findings could be the shared time reference of questionnaires and clinician ratings, as both, unlike EMA, rely on retrospective recall.

### 4.3. Additional Analysis

In light of these results, we conducted additional analyses and discovered significant differences in the time effects (slopes) of EMA and WQA. The WQA scores exhibited a steeper decrease over time, with more extreme scores in both directions—higher at baseline and lower postintervention—compared to the EMA scores. The effect was evident for changes in RNT and depressive symptoms, as well as at the level of individual RNT items that were identical between EMA and WQA. This observation indicates that the effect is not solely attributable to differences in item selection between the EMA and WQA scores of depressive symptoms and RNT. A plausible explanation for these findings could be the presumed recall bias associated with questionnaires: while in the past, it was predominantly assumed that memories of depressed patients are negatively biased [46], more recent literature suggests that memories exaggerate reality in both negative and positive valence. This bias is amplified in depressed patients—stronger in terms of negative than positive bias, but still in both directions [9]. Theoretically, this effect could lead to overestimations of time effects in depression trials when relying on retrospective questionnaires. As depressed individuals are more susceptible to recall bias than healthy individuals, it is plausible that retrospectively they may overestimate the severity of their symptoms at the beginning of a therapy, while when they are less depressed at the end of a therapy, their retrospective self-reports might be more realistic or even positively biased. Our study supports this assumption. Nevertheless, alternative explanations for these findings cannot be ruled out. For instance, it is plausible that EMA might exhibit smaller change amplitudes over time due to anchoring effects. When individuals are frequently rating the same items, as with EMA, previous responses may serve as reference points, influencing subsequent answers. Consequently, patients' EMA ratings may exhibit high interdependence, whereas their questionnaire ratings, collected at 1-week intervals and containing a larger number of items, may elicit more independent responses.

## 5. Conclusion

Aligned with prior research, our study establishes significant associations among clinical interview, questionnaires, and EMA ratings of change in depression. Despite substantial evidence supporting EMA's ability to detect higher effect sizes between intervention conditions than questionnaires, our findings, unlike Moore et al. [20], suggest a more nuanced perspective. The assumption that EMA is simply more sensitive to change falters, as our study indicates smaller change amplitudes in EMA compared to questionnaires. Therefore, an alternative perspective emerges, suggesting that EMA provides more accurate estimates, enhancing statistical power and resulting in clearer effects, as indicated by a recent simulation study [47]. Sampling and/or memory biases, which are inherent in depressed patients, may undergo changes during therapy, thereby systematically influencing the repeated assessments of retrospective questionnaires. Especially, when investigating intervention effects in modest sample sizes, coupled with active control conditions, which lower the expected effect size differences, this systematic bias may hinder the detection of significant effects. Therefore, we assume that it is not EMA's heightened sensitivity to symptom change but its lower levels of sampling and memory biases making it more sensitive to intervention effects.

Nevertheless, our study prompts questions about what each instrument truely measures and which is most valid for monitoring depression-related change. Beyond recall bias, factors like current mood or expectations introduce biases into retrospective self-reports. While clinical interviews aim to mitigate such biases, it is uncertain that if they are free of them and their nature of external ratings introduce other potential biases, such as influences of the rater on the social desirability of the interviewee. It can be assumed that the momentary nature of EMA inbedded in the natural environment of the patient avoids these biases, but being asked the same question several times per day introduces risks of low conscientiousness or anchoring effects. Another perspective is to consider questionnaires more as a trait measure and EMA more as a state measure of depression. The EMA in our study sampled momentary states (“right now” states), whereas the questionnaires captured an aggregated subjective measure of depression over time (e.g., 2 weeks in the case of BDI-II). Complementing questionnaires with EMA could therefore become a progressive approach that allows the investigation of three distinct aspects: the momentary change in depressive symptoms, patients' perceived change, and the discrepancy between the two as a reflection of change in memory bias. In conclusion, all three assessment techniques—EMA, questionnaires and clinical interviews—pose distinct strengths, but also biases challenging their validity. Therefore, it might be promising to zoom out further and consider not retrospectivity but subjectivity as the fundamental problem of self-ratings. On this point, questionnaires, clinical interviews, and even EMA ratings have their limits, as they are all based on self-reports. Therefore, it might be a promising avenue to develop more creative EMA approaches that combine self-reports with objectively measured behavioral markers of depression, such as homestay, social avoidance, physical activity, and sleep [48].

## Figures and Tables

**Table 1:** Descriptive statistics of the treatment arms.

Characteristic	Total		Treatment						*t* or *χ*^2^	df	*p*
			ST		CBT		IST				
	(*N* = 71)		(*N* = 20)		(*N* = 28)		(*N* = 23)				
	*N*	Percentage	*N*	Percentage	*N*	Percentage	*N*	Percentage			
Gender (female)	39	54.93	15	75.00	10	35.71	14	60.87	7.65	2	0.02
Nationality (German)	60	84.51	16	80.00	25	89.29	19	82.61	0.77	2	0.68
School graduation (qualification for university entrance)	42	59.15	11	55.00	19	67.86	12	52.17	1.80	2	0.41
Income	—	—	—	—	—	—	—	—	5.74	2	0.06
Low income (up to 1,500 EUR)	29	40.85	6	30.00	15	53.57	8	34.78	—	—	—
Middle income (1,500–4,000 EUR)	27	38.03	10	50.00	6	21.43	11	47.83	—	—	—
High income (more than 4,000 EUR)	11	15.49	3	15.00	7	25.00	1	4.35	—	—	—
Not specified	4	5.63	0	0.00	0	0.00	3	13.04	—	—	—
	Mean	SD	Mean	SD	Mean	SD	Mean	SD	*t* or *χ*^2^	df	*p*

Age (years)	40.96	12.17	41.35	11.44	38.54	13.65	43.57	10.71	2.07	2	0.36
Response rates											
EMA	57.77	25.31	57.92	27.22	62.56	24.54	51.81	24.34	2.59	2	0.27
BDI	92.08	13.57	92.50	13.08	93.75	11.02	89.67	16.71	1.03	2	0.60
PTQ	90.49	14.86	91.88	13	91.96	14.52	87.50	16.85	2.33	2	0.31
Baseline symptoms											
BDI	32.56	8.40	33.80	7.98	29	7.35	35.83	8.62	4.99	2	0.01
PTQ	40.44	11.19	41	12.44	36.54	11.96	44.70	7.11	6.11	2	0.05
WHODAS	2.87	0.65	2.92	0.56	2.67	0.67	3.08	0.67	2.61	2	0.08

*Note*. ST, schema therapy; CBT, cognitive behavioral therapy; IST, individual supportive therapy; BDI-II; Beck's Depression Inventory-II; PTQ, Perseverative Thinking Questionnaire; and WHO-DAS, World Health Organization-Disability Assessment Schedule.

**Table 2:** Multilevel models of depression and RNT measured by EMA versus WQA among patients randomly assigned to ST, IST, or CBT, with ST as reference group.

DV/predictors	Estimates	SE	*t* (*p*)	95% CI
EMA–depressive symptoms				
Intercept	0.35	0.11	3.06 (0.002)	[0.13–0.57]
Condition (CBT–ST)	−0.26	0.15	−1.77 (0.082)	[−0.56 to 0.03]
Condition (IST–ST)	−0.12	0.16	−0.75 (0.459)	[−0.43 to 0.2]
Time	<0.01	<0.01	−3.03 (0.002)	[−0.01 to <0.00]
Time × condition (CBT–ST)	<0.01	<0.01	1.78 (0.076)	[ <0.00–0.01]
Time × condition (IST–ST)	<0.01	<0.01	0.78 (0.437)	[ <0.00–0.01]
WQA–depressive symptoms				
Intercept	1.08	0.14	7.78 (<0.001)	[0.81–1.35]
Condition (CBT–ST)	−0.14	0.18	−0.75 (0.454)	[−0.5 to 0.22]
Condition (IST–ST)	−0.04	0.19	−0.23 (0.818)	[−0.42 to 0.33]
Time	−0.32	0.04	−8.41 (<0.001)	[−0.39 to −0.24]
Time × condition (CBT–ST)	0.05	0.05	0.99 (0.32)	[−0.05 to 0.15]
Time × condition (IST–ST)	0.01	0.05	0.21 (0.836)	[−0.09 to 0.11]
EMA–RNT				
Intercept	0.52	0.10	5.15 (<0.001)	[0.32–0.71]
Condition (CBT–ST)	−0.47	0.13	−3.59 (0.001)	[−0.73 to −0.21]
Condition (IST–ST)	−0.33	0.14	−2.37 (0.021)	[−0.6 to −0.05]
Time	−0.01	<0.01	−5.33 (<0.001)	[−0.01 to <0.01]
Time × condition (CBT–ST)	0.01	<0.01	3.75 (<0.001)	[ <0.01–0.01]
Time × condition (IST–ST)	<0.01	<0.01	2.46 (0.014)	[ <0.01–0.01]
WQA–RNT				
Intercept	0.84	0.18	4.64 (<0.001)	[0.48–1.19]
Condition (CBT–ST)	−0.12	0.24	−0.5 (0.62)	[−0.59 to 0.35]
Condition (IST–ST)	−0.21	0.25	−0.87 (0.387)	[−0.7 to 0.27]
Time	−0.25	0.05	−4.88 (<0.001)	[−0.34 to −0.15]
Time × condition (CBT–ST)	0.04	0.07	0.62 (0.538)	[−0.09 to 0.17]
Time × condition (IST–ST)	0.06	0.07	0.8 (0.425)	[−0.08 to 0.19]

*Note*. EMA, ecological momentary assessment; WQA, weekly questionnaire assessment; ST, schema therapy; CBT, cognitive behavioral therapy; IST, individual supportive therapy; and RNT, repetitive negative thinking. Sample size in all models is 71.

**Table 3:** Standardized effect sizes (Cohen's *d* and NNT) of depression and RNT measured by EMA versus WQA among patients randomly assigned to ST, IST, or CBT.

Variables/group comparisons	Cohen's *d*	NNT
EMA–depressive symptoms		
IST–ST	0.26	10
CBT–ST	0.62	4
WQA–depressive symptoms		
IST–ST	0.1	24
CBT–ST	0.47	5
EMA–RNT		
IST–ST	0.87	3
CBT–ST	1.46	2
WQA–RNT		
IST–ST	0.35	7
CBT–ST	0.26	10

*Note*. EMA, ecological momentary assessment; WQA, weekly questionnaire assessment; ST, schema therapy; CBT, cognitive behavioral therapy; IST, individual supportive therapy; RNT, repetitive negative thinking; and NNT, number-needed-to-treat. Sample size in all analyses is 71.

**Table 4:** Multiple regression analyses predicting global functioning (GF) measured with clinical interview (WHO-DAS) after 7 weeks of treatment based on baseline global functioning and the slope of depression and RNT measured with EMA versus WQA.

DV/predictors	Estimates	SE	*t* (p)	95% CI	*F* statistic	*R* ^2^
EMA–depressive symptoms						
Intercept	0.55	0.24	2.34 (0.022)	[0.08–1.02]	*F* (2.68) = 30.52 (<0.001)	0.47
GFpre	0.60	0.08	7.46 (<0.001)	[0.44–0.76]		
Slope (EMA–dep)	26.13	9.32	2.8 (0.007)	[7.54–44.72]		
WQA–depressive symptoms						
Intercept	1.07	0.27	3.99 (<0.001)	[0.54–1.61]	*F* (2.68) = 35.92 (<0.001)	0.51
GFpre	0.58	0.08	7.54 (<0.001)	[0.43–0.73]		
Slope (WQA–dep)	1.80	0.48	3.77 (<0.001)	[0.85–2.76]		
EMA and WQA–depressive symptoms						
Intercept	0.99	0.27	3.61 (0.001)	[0.44–1.53]	*F* (3.67) = 25.04 (<0.001)	0.53
GFpre	0.59	0.08	7.69 (<0.001)	[0.44–0.74]		
Slope (EMA–dep)	14.28	9.83	1.45 (0.151)	[−5.34 to 33.89]		
Slope (WQA–dep)	1.48	0.53	2.81 (0.006)	[0.43–2.53]		
EMA–RNT						
Intercept	0.57	0.24	2.33 (0.023)	[0.08–1.05]	*F* (2.68) = 27.18 (<0.001)	0.44
GFpre	0.59	0.08	7.19 (<0.001)	[0.43–0.76]		
Slope (EMA–RNT)	21.48	10.81	1.99 (0.051)	[−0.09 to 43.05]		
WQA–RNT						
Intercept	0.86	0.25	3.45 (0.001)	[0.36–1.36]	*F* (2.68) = 33.83 (<0.001)	0.5
GFpre	0.55	0.08	6.95 (<0.001)	[0.39–0.71]		
Slope (WQA–RNT)	1.09	0.32	3.43 (0.001)	[0.46–1.72]		
EMA and WQA–RNT						
Intercept	0.86	0.26	3.34 (0.001)	[0.35–1.37]	*F* (3.67) = 22.24 (<0.001)	0.5
GFpre	0.55	0.08	6.85 (<0.001)	[0.39–0.71]		
Slope (EMA–RNT)	1.83	12.64	0.15 (0.885)	[−23.4 to 27.06]		
Slope (WQA–RNT)	1.06	0.39	2.7 (0.009)	[0.28–1.84]		

*Note*. RNT, repetitive negative thinking; dep, depression; GF, global functioning; EMA, ecological momentary assessment; and WQA, weekly questionnaire assessment. All models have two degrees of freedom. Sample size in all models is 71.

**Table 5:** Multilevel models of depression and RNT measured by EMA versus WQA with assessment technique (EMA versus WQA) as predictor.

DV/predictors	Estimates	SE	*t* (or *z*)	95% CI
Depressive symptoms				
Intercept	0.29	0.09	3.37 (0.001)	[0.12–0.46]
Time	−0.09	0.02	−3.57 (<0.001)	[−0.13 to −0.04]
Assessment technique	0.72	0.09	8.28 (<0.001)	[0.55–0.89]
Assessment technique × time	−0.21	0.02	−9.74 (<0.001)	[−0.25 to −0.16]
RNT				
Intercept	0.37	0.10	3.82 (<0.001)	[0.18–0.55]
Time	−0.11	0.03	−3.99 (<0.001)	[−0.16 to −0.06]
Assessment technique	0.36	0.09	3.92 (<0.001)	[0.18–0.53]
Assessment technique × time	−0.10	0.02	−4.65 (<0.001)	[−0.15 to −0.06]
RNT item “repetitiveness”				
Intercept	0.38	0.09	4.3 (<0.001)	[0.21–0.56]
Time	−0.11	0.02	−4.62 (<0.001)	[−0.16 to −0.07]
Assessment technique	0.26	0.10	2.69 (0.007)	[0.07–0.45]
Assessment technique × time	−0.08	0.02	−3.21 (0.001)	[−0.12 to −0.03]
RNT item “intrusiveness”				
Intercept	0.35	0.09	3.8 (<0.001)	[0.17–0.53]
Time	−0.10	0.03	−4.08 (<0.001)	[−0.15 to −0.05]
Assessment technique	0.20	0.10	2.06 (0.04)	[0.01–0.39]
Assessment technique × time	−0.06	0.02	−2.46 (0.014)	[−0.11 to −0.01]
RNT item “controllability”				
Intercept	0.33	0.09	3.57 (<0.001)	[0.15–0.51]
Time	−0.10	0.03	−3.87 (<0.001)	[−0.15 to −0.05]
Assessment technique	0.23	0.10	2.32 (0.021)	[0.04–0.42]
Assessment technique × time	−0.06	0.02	−2.71 (0.007)	[−0.11 to −0.02]

*Note*. EMA, ecological momentary assessment; WQA, weekly questionnaire assessment; dep, depression; and RNT, repetitive negative thinking. Sample size in all models is 71.

## Data Availability

Due to the protection of participants' rights to privacy and data protection, the dataset, the materials, and the analysis code are not publicly available but are available by emailing the corresponding author.

## Abbreviations

CBT: Cognitive behavioral therapy
EMA: Ecological momentary assessment
IST: Individual supportive therapy
NNT: Number needed to treat
OPTIMA: Optimized psychotherapy identification at the Max-Planck-Institute of Psychiatry
RCT: Randomized controlled trial
ST: Schema therapy
WQA: Weekly questionnaire assessment
LRM: Linear regression model
MLM: Multilevel model.

## Additional Points

*Limitations*. This study has several limitations. To our knowledge, this was the first study comparing EMA and WQA monitoring of depression-related constructs using different items in terms of quantity and wording. While this approach increases the external validity of our results, it also lowers the comparability between our EMA and WQA outcomes compared to previous studies. A 1 : 1 transfer of questionnaires into an EMA setting is difficult, as questionnaires usually have a substantial number of items. Administering such a high item count on a daily basis or multiple times per day could overly burden patients. Previous studies have therefore compared questionnaire subscales between EMA and questionnaires, which deviates from the standard of clinical trials, where full questionnaires are usually administered. In our additional analysis, however, we could demonstrate that significant differences between EMA and WQA slopes persist even at the level of identical items. Additionally, it is essential to consider that clinical interviews might not serve as the best benchmark for comparing the predictive validity of EMA and WQA. Both, questionnaires and clinical interviews collect symptoms retrospectively, carrying the risk of recall biases. Even though clinical interviews are third-party ratings conducted by trained personnel, which should reduce this risk [49], the retrospectivity of both assessment methods may explain why questionnaires predict the results of clinical interviews stronger than EMA.

Furthermore, our sample exhibited variations across the three intervention conditions in terms of demographical and clinical aspects. Despite our efforts to control for these variables in our analyses, the inherent heterogeneity of the sample, coupled with its modest size and the fact that psychotherapy was an important, but just a part of the comprehensive treatment program, compounds the interpretation of effects between conditions. Additionally, the monocentric study design, in which most patients were from the area of Munich, Germany, limits the generalizability of our study due to selection bias.

## Supplementary Materials

Supplementary Materials The supplementary materials include an overview of the assessment plan, the wordings of the EMA items, further descriptives of the study sample, comparison analyses of simple and complex MLMs including all covariates, survival analyses of dropout and EMA response rate distributions across treatment weeks.
