# Supplementary material for: Ecological Momentary Assessment versus Weekly Questionnaire Assessment of Change in Depression
Source: Depress Anxiety. 2024 Jul 11;2024:9191823. doi: 10.1155/2024/9191823 (PMC11919112; doi:10.1155/2024/9191823)
Supplement: Supplementary Materials — The supplementary materials include an overview of the assessment plan, the wordings of the EMA items, further descriptives of the study sample, comparison analyses of simple and complex MLMs including all covariates, survival analyses of dropout and EMA response rate distributions across treatment weeks. [file 9191823.f1.docx]

The supplementary material provided here is intended to offer readers a more comprehensive view of the study and to ensure full transparency in the presentation of our research findings. In line with the submission guidelines, we have included six key components that are referenced in the main manuscript:

- **Supplementary Table 1:** This table provides an overview over the assessment plan of the study.
- **Supplementary Table 2:** This table provides the precise wordings of each individual EMA (Ecological Momentary Assessment) item used in our study.
- **Supplementary Table 3:** This table provides an overview over further descriptives of the sample, such as care condiction, comorbidities and co-therapies.
- **Supplementary Table 4:** This table provides the results of the comparison analyses conducted between the simple multilevel models, which included only time and intervention condition as predictors and the complex multilevel models, which were expanded to include the covariates gender, baseline depression, response rate and concomitant care.
- **Supplementary Figure 1:** This figure visually represents the outcomes of a survival analysis conducted to assess whether dropout rates were evenly distributed across all intervention groups. This analysis is an essential aspect of understanding the dropout patterns in our study and its potential impact on the overall findings.
- **Supplementary Figure 2:** This figure shows the distribution of patient response rates to the ecological momentary assessment (EMA) over the intervention weeks.

For a comprehensive understanding of the study, we encourage readers to refer to these supplementary materials when prompted in the main manuscript. We believe that they significantly contribute to the overall transparency and depth of the research presented.

## **Supplementary Table 1**

*Assessment Plan of the Study*

|  | | Baseline week | | | | | | | Week 1 - 6 | | | | | | | Week 7 | | | | | | |
| --- | --- | --- | --- | --- | --- | --- | --- | --- | --- | --- | --- | --- | --- | --- | --- | --- | --- | --- | --- | --- | --- | --- |
| Weekdays | | 1 | 2 | 3 | 4 | 5 | 6 | 7 | 1 | 2 | 3 | 4 | 5 | 6 | 7 | 1 | 2 | 3 | 4 | 5 | 6 | 7 |
| Intervention (CBT/ST/IST) | |  | | | | | | | 2x single- &  2x group-sessions | | | | | | | 2x single- &  2x group-sessions | | | | | | |
| Assessments | |  |  |  |  |  |  |  |  |  |  |  |  |  |  |  |  |  |  |  |  |  |
|  | Eligibility check | X | | | | | | |  | | | | | | |  | | | | | | |
|  | EMA | 3x | 3x | 3x | 3x | 3x | 3x | 3x | 3x | 3x | 3x | 3x | 3x | 3x | 3x | 3x | 3x | 3x | 3x | 3x | 3x | 3x |
|  | BDI-II/ PTQ | X | | | | | | | X | | | | | | | X | | | | | | |
|  | WHODAS | X | | | | | | |  | | | | | | | X | | | | | | |

*Note*. This table gives a timeline of the interventions and assessments in the study. Note that only those assessments relevant for our analyses are mentioned here. CBT: Cognitive Behavioral Therapy; ST: Schema Therapy; IST: Individual Supportive Therapy; EMA: Ecological Momentary Assessment; BDI-II; Beck’s Depression Inventory II; PTQ: Perseverative Thinking Questionnaire; WHO-DAS: World Health Organization Disability Assessment Schedule.

## **Supplementary Table 2**

*Wordings of the EMA Items*

| EMA Item construct | **Item Wording (German original)** | **Item Wording (English translation)** |
| --- | --- | --- |
| **Depressive symptoms** |  |  |
| Loss of interest | Hast du gerade das Gefühl, zu nichts mehr Lust zu haben? | Do you feel like you don't want to do anything anymore? |
| Withdrawal | Ziehst du dich gerade von sozialen Kontakten oder Aktivitäten zurück? | Are you currently withdrawing from social contacts or activities? |
| Psychomotor agitation /inhibition | Fühlst du dich gerade besonders körperlich gehemmt oder aktiviert? | Are you feeling particularly physically inhibited or agitated? |
| Current mood | Wie fühlst du dich? | How are you feeling? |
| **RNT** |  |  |
| Repetitiveness of RNT | Dieselben negative Gedanken gehen mir immer und immer wieder durch den Kopf. | The same negative thoughts keep going through my mind again and again. |
| Uncontrollability of RNT | Ich hänge an bestimmten negative Gedanken fest und kann mich nicht davon lösen. | I get stuck on certain negative issues and can’t move on. |
| Intrusiveness RNT | Negative Gedanken tauchen auf, ohne dass ich dies will. | Negative thoughts come to my mind without me wanting them to. |
| Subjective burden through RNT | Ich fühle mich durch negative Gedanken beeinträchtigt. | I feel weighted down by negative thoughts. |

*Note.* The response scale of all EMA Items, except for the mood item, was two-stepped. Participants responded to a binary *Yes-No* scale. If *Yes* was selected, a five-point Likert scale followed, which assessed the extent of agreement (labeling: *not at all, a bit, moderately, considerably, very much*). The ‘Current mood’ item was rated by selecting one of five emojis (labeling: *very good*, *good*, *moderate*, *bad*, *very bad*).

## **Supplementary Table 3**

*Descriptive Statistics of the Treatment Arms*

|  |  |  | **Treatment** | | | | | |  |  |  |
| --- | --- | --- | --- | --- | --- | --- | --- | --- | --- | --- | --- |
|  | **Total** | | **ST** | | **CBT** | | **IST** | |  |  |  |
| **Characteristic** | (N=71) | | (N=20) | | (N=28) | | (N=23) | |  |  |  |
|  | **N** | **%** | **N** | **%** | **N** | **%** | **N** | **%** | **t or Chi^2^** | **df** | ***p*** |
| **Martital status** (married or steady relationship) | 31 | 43.66 | 9 | 45.00 | 15 | 53.57 | 7 | 30.43 | 2.59 | 2 | 0.27 |
| **Care condition** |  |  |  |  |  |  |  |  | 6.54 | 2 | 0.04 |
| Inpatient care | 46 | 64.79 | 9 | 45.00 | 18 | 64.29 | 19 | 82.61 |  |  |  |
| Day clinical care | 25 | 35.21 | 11 | 55.00 | 10 | 35.71 | 4 | 17.39 |  |  |  |
| **Covid-19**^a^ |  |  |  |  |  |  |  |  | 0.92 | 2 | 0.63 |
| Before Corona | 21 | 29.58 | 5 | 25.00 | 7 | 25.00 | 9 | 39.13 |  |  |  |
| Partly during Corona | 10 | 14.08 | 2 | 10.00 | 6 | 21.43 | 2 | 8.70 |  |  |  |
| During Corona | 40 | 56.34 | 13 | 65.00 | 15 | 53.57 | 12 | 52.17 |  |  |  |
| **Co-therapies** |  |  |  |  |  |  |  |  |  |  |  |
| Ergotherapy | 59 | 83.10 | 18 | 90.00 | 24 | 85.71 | 17 | 73.91 | 2.17 | 2 | 0.34 |
| Case management | 62 | 87.32 | 16 | 80.00 | 25 | 89.29 | 21 | 91.30 | 1.38 | 2 | 0.50 |
| Relaxation training | 26 | 36.62 | 6 | 30.00 | 11 | 39.29 | 9 | 39.13 | 0.52 | 2 | 0.77 |
| Cognitive training | 2 | 2.82 | 1 | 5.00 | 1 | 3.57 | 0 | 0.00 | 1.06 | 2 | 0.59 |
| Sports | 35 | 49.30 | 6 | 30.00 | 15 | 53.57 | 14 | 60.87 | 4.35 | 2 | 0.11 |
| **Axis I comorbidities^b^** |  |  |  |  |  |  |  |  |  |  |  |
| Agoraphobia | 3 | 4.23 | 2 | 11.11 | 0 | 0.00 | 1 | 4.76 | 0.19 | 2 | 0.91 |
| Panic disorder | 7 | 9.86 | 2 | 11.11 | 4 | 14.81 | 1 | 4.76 | 1.81 | 2 | 0.40 |
| Panic disorder with agoraphobia | 8 | 11.27 | 1 | 5.56 | 3 | 11.11 | 4 | 19.05 | 1.32 | 2 | 0.52 |
| Social phobia | 15 | 21.13 | 4 | 22.22 | 5 | 18.52 | 6 | 28.57 | 0.23 | 2 | 0.89 |
| Specific phobia | 15 | 21.13 | 5 | 27.78 | 5 | 18.52 | 5 | 23.81 | 0.02 | 2 | 0.99 |
| Generalized anxiety disorder | 9 | 12.68 | 2 | 11.11 | 2 | 7.41 | 5 | 23.81 | 1.08 | 2 | 0.58 |
| Post-traumatic stress disorder | 12 | 16.90 | 5 | 27.78 | 2 | 7.41 | 5 | 23.81 | 0.97 | 2 | 0.62 |
| Obsessive compulsive disorder | 6 | 8.45 | 1 | 5.56 | 2 | 7.41 | 3 | 14.29 | 0.73 | 2 | 0.69 |
| Eating Disorder | 3 | 4.23 | 1 | 5.56 | 0 | 0.00 | 2 | 9.52 | 0.25 | 2 | 0.88 |
| Substance disorder | 13 | 18.31 | 5 | 27.78 | 2 | 7.41 | 6 | 28.57 | 1.37 | 2 | 0.50 |
| Somatoform disorder | 19 | 26.76 | 8 | 44.44 | 3 | 11.11 | 8 | 38.10 | 3.10 | 2 | 0.21 |
|  | **Mean** | **SD** | **Mean** | **SD** | **Mean** | **SD** | **Mean** | **SD** | **t or Chi^2^** | **df** | ***p*** |
| **Medication** (no. of weeks) |  |  |  |  |  |  |  |  |  |  |  |
| Antidepressants | 6.56 | 2.69 | 6.20 | 3.19 | 6.04 | 3.01 | 7.52 | 1.31 | 4.22 | 2 | 0.12 |
| Neuroleptics | 1.83 | 3.18 | 0.80 | 2.46 | 2 | 3.27 | 2.52 | 3.51 | 4.12 | 2 | 0.13 |
| Tranquilizer | 0.80 | 2.09 | 0.90 | 2.27 | 1.18 | 2.45 | 0.26 | 1.25 | 2.95 | 2 | 0.23 |
| Mood Stabilizer | 0.34 | 1.44 | 0.05 | 0.22 | 0.54 | 1.73 | 0.35 | 1.67 | 0.96 | 2 | 0.62 |

*Note*. ST: Schema Therapy; CBT: Cognitive Behavioral Therapy; IST: Individual Supportive Therapy; BDI-II; Beck’s Depression Inventory II; PTQ: Perseverative Thinking Questionnaire; WHO-DAS: World Health Organization Disability Assessment Schedule.

^a^The start of the Covid-19 pandemic was set at 10th March 2020, coinciding with the enforcement of initial hygiene measures and mandatory visiting restrictions for staff and patients in our clinic.

^b^Comorbidities were diagnosed by the Munich-Composite International Diagnostic Interview (M-CIDI; Wittchen et al., 1998), which is a computerized, fully standardized German version of the World Mental Health Composite Internatinal Diagnostic Interview (WHO-CIDI; Robins et al. 1988).

## **Supplementary Table 4**

*Comparison Analyses between simple and complex Multilevel Models*

| Model | df | AIC | BIC |
| --- | --- | --- | --- |
| **EMA – Depressive symptoms** |  |  |  |
| Simple MLM | 10 | 19038.21 | 19106.59 |
| Complex MLM | 27 | 19067.14 | 19251.76 |
| **WQA – Depressive symptoms** |  |  |  |
| Simple MLM | 10 | 1119.69 | 1162.111 |
| Complex MLM | 27 | 1150.25 | 1264.794 |
| **EMA – RNT** |  |  |  |
| Simple MLM | 10 | 19109.75 | 19178.13 |
| Complex MLM | 27 | 19138.50 | 19323.13 |
| **WQA – RNT** |  |  |  |
| Simple MLM | 10 | 1276.71 | 1319.14 |
| Complex MLM | 27 | 1307.45 | 1421.99 |

*Note*. This table provides the results of the comparison analyses conducted between the simple multilevel models, which included only time and intervention condition as predictors and the complex multilevel models in those the covariates gender, baseline depression, response rate and concomitant care were added. RNT: Repetitive Negative Thinking; EMA: Ecological Momentary Assessment; WQA: Weekly Questionnaire Assessment; MLM: Multi Level Model.

## **Supplementary Figure 1**

*Proportion of Patients in the Treatment Arms (ST, IST, or CBT) throughout the Baseline Week and the seven-week Treatment Phase*


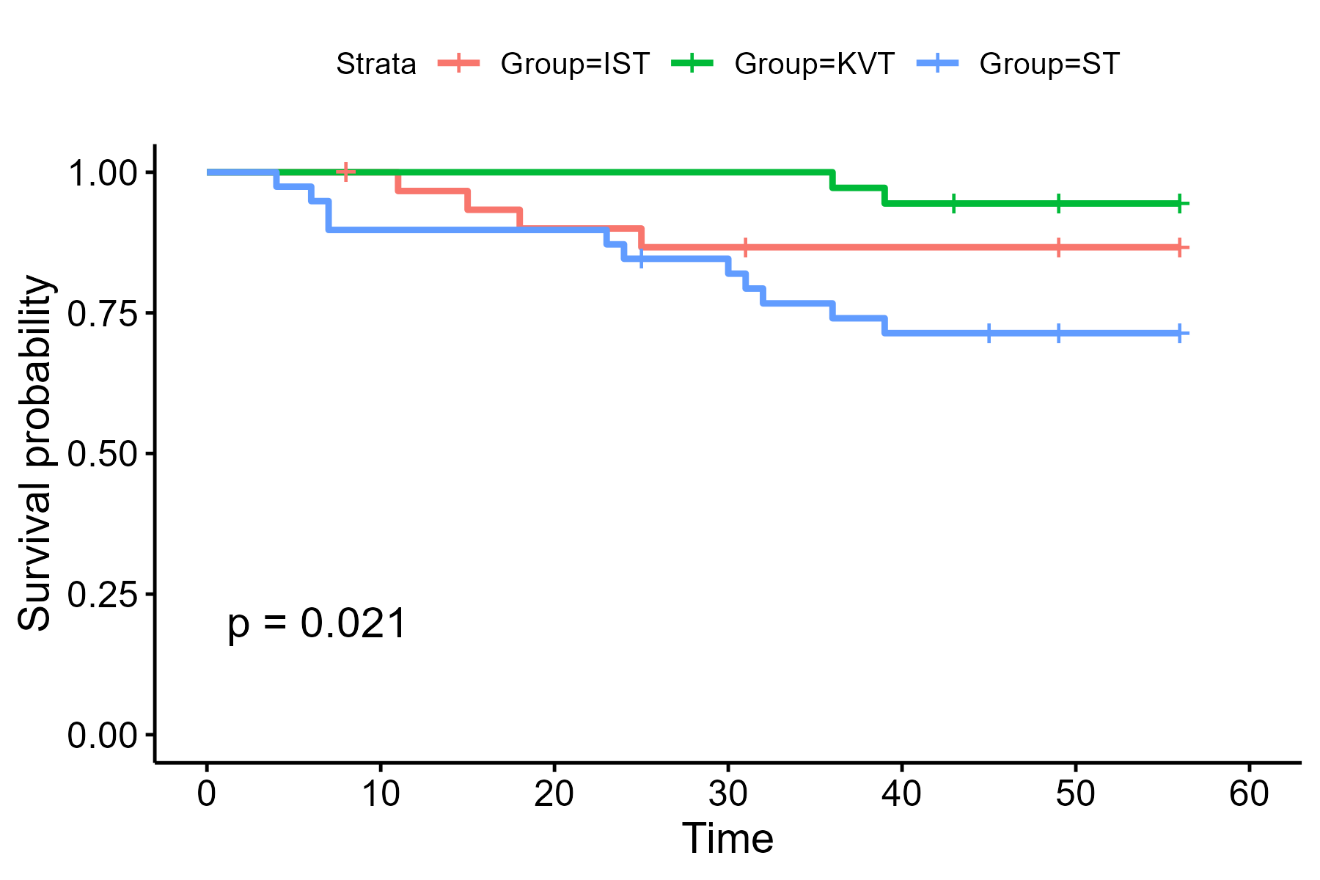


*Note*. N = 106 (Intent-to-treat sample); ST: Schema Therapy; CBT: Cognitive Behavioral Therapy; IST: Individual Supportive Therapy; Drop-outs during the study were defined as enrolled patients who left the clinic before end of intervention, who missed more than six sessions of their intervention (equivalent to 22% of the total intervention dose) or requested for a different treatment.

## **Supplementary Figure 2**

*Distribution of Patient Response rates to the Ecological Momentary Assessment over the intervention weeks*


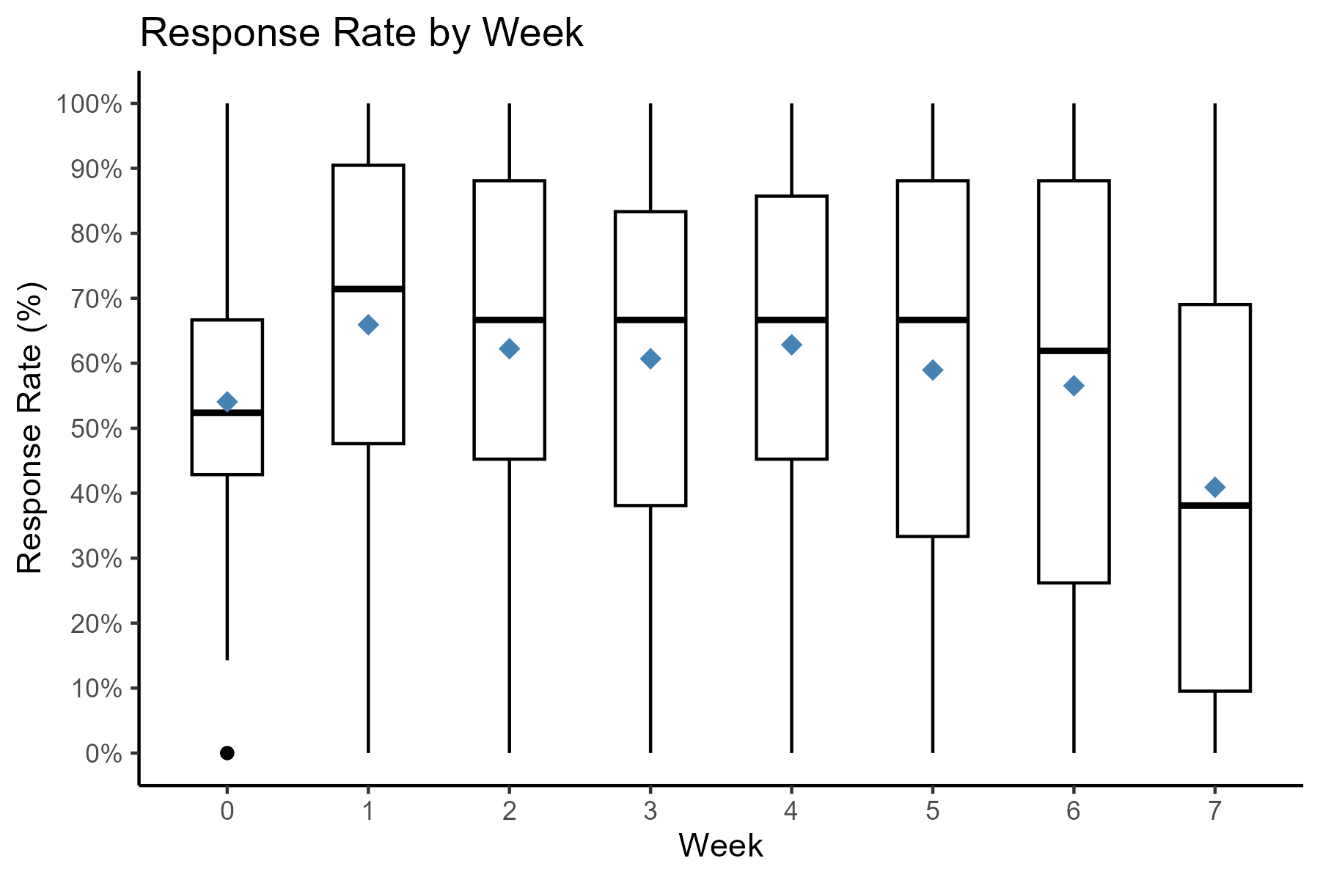


*Note*. N = 71; Week: Intervention week; Comparison of patient response rates to the ecological momentary assessment over the intervention weeks. The boxplots show the distribution of the response rates for each treatment week. The box represents the interquartile range (IQR), with the line inside representing the median and the blue square representing the mean response rate. The whiskers extend to the minimum and maximum values within 1.5 times the IQR above the quartiles. Outliers are represented by individual points outside the whiskers.

**References**

Robins LN, Wing J, Wittchen HU, Helzer JE, Babor TF, Burke J, et al. The Composite International Diagnostic Interview. An epidemiologic Instrument suitable for use in conjunction with different diagnostic systems and in different cultures. *Arch Gen Psychiatry*. 1988 Dec;45(12):1069–77. <https://doi.org/10.1001/archpsyc.1988.01800360017003>

Wittchen HU, Lachner G, Wunderlich U, Pfister H. Test-retest reliability of the computerized DSM-IV version of the Munich-Composite International Diagnostic Interview (M-CIDI). *Soc Psychiatry Psychiatr Epidemiol*. 1998 Oct 1;33(11):568–78. <https://doi.org/10.1007/s001270050095>
